# The effect of mind-body exercise on blood pressure in middle-aged and elderly patients with hypertension: A protocol for a systematic review and meta-analysis

**DOI:** 10.1097/MD.0000000000026452

**Published:** 2021-06-25

**Authors:** Beihai Ge, Hao Chen, Xianhui Liao

**Affiliations:** a Department of Neurology, Guangxi Zhuang Autonomous Region Brain Hospital, Liuzhou, Guangxi; b Institute of Physical Education and International Equestrian, Wuhan Business University; c Department of Sports, Wuhan EQ & IQ School, Wuhan, Hubei, China.

**Keywords:** blood pressure, hypertension, meta-analysis, middle-aged and elderly patients, mind-body exercise, protocol

## Abstract

**Background:**

Depending on the person, cervical spondylosis may have no clinical symptoms, but cervical spondylosis will definitely cause changes in people's blood pressure, which will further affect physical and mental health.

**Objectives:**

This study aims to explore the effect and safety of mind-body exercise intervention on the blood pressure in middle-aged and elderly patients with hypertension through meta-analysis.

**Methods:**

This meta-analysis searched studies from 4 research databases: the China National Knowledge Infrastructure (from 1979), Web of Science (from 1950), PubMed (from 1965), and Cochrane (from 1991), Date of retrieval: January 22, 2021, Two authors will independently search literature records, scan titles, abstracts, and full texts, collect data, and assess materials for risk of bias. The data will be analyzed by Stata 14.0 software.

**Results:**

The present study is a systematic review and meta-analysis program with no results. Data analysis will be completed after the program has been completed.

**Discussion:**

This meta-analysis may provide clinical practice with more reliable evidence-based medical evidence that mind-body exercise can benefit the blood pressure of middle-aged and elderly hypertensive patients.

**INPLASY Registration Number:**

INPLASY202130072.

## 1 Introduction

The symptoms of hypertension vary from person to person. There may be no symptoms or symptoms that are not obvious in the early stage, and there will be occasional symptoms such as fatigue and palpitation, but these conditions generally only occur after fatigue, mental tension, and mood-swings and can quickly return to normal following rest.^[1]^ However, the chronic effects of hypertension often slowly deteriorate people's health.^[2]^ Studies have demonstrated that there is a certain correlation between the symptoms of hypertension and the level of blood pressure. When hypertension symptoms are serious, confusion and convulsions will occur, which is not difficult to cause irreversible pathological changes and damage to the heart, brain, kidney, and other target organs in a short time.^[3]^ Data from the *Lancet* showed that the number of patients with hypertension in the world had exceeded 1.1 billion, seriously endangering people's health.^[4]^ Ezzati et al of the School of Public Health at Imperial College, UK, stated: “High blood pressure was a great risk factor for stroke and heart disease, with about 7.5 million deaths worldwide each year.”^[5]^ Therefore, how to actively treat hypertension is worthy of research and discussion in this century.

Mind-body exercise originates from the East and includes Taijiquan, Baduanjin, and Qigong, among others. It has been proven that mind-body exercise is beneficial to health and can improve happiness and satisfaction.^[6]^ In addition, studies have proved that Baduanjin and Qigong have positive effects on blood pressure in patients with hypertension, which provides a specified reference basis. However, the subjects’ ages in these studies are between 18 and 75.^[7]^ According to the clinical observation, middle-aged and elderly people are the multi-incidence patient populations with hypertension,^[8]^ Despite this, the existing research results do not have the related research characteristics aimed at this population. However, Mind-body exercise, as low-cost exercise therapy, may be a good choice for middle-aged and elderly patients with hypertension.

Compared with traditional experimental research, meta-analysis is a systematic review method with a combination of quantitative and qualitative analysis, which contributes to expand the sample size of this research topic and obtain more objective and scientific results.^[9]^ The existing research takes mind-body exercise (Taijiquan, Baduanjin, and Qigong) as a scheme to improve blood pressure, but the existing research has not reached a unified consensus among the related results of research design, exercise time, exercise frequency, exercise volume, and other variables, which is not conducive to the wide range of Mind-body exercise to the treatment of patients with hypertension. The purpose of this study is to evaluate the efficacy and safety of mind-body exercise in improving blood pressure in middle-aged and elderly patients with hypertension and to provide operative suggestions for middle-aged and elderly patients with hypertension and clinicians to improve blood pressure.

## 2 Methods

### 2.1 Search strategy and registration

In this study, the following research databases were searched: the China National Knowledge Infrastructure (from 1979), Web of Science (from 1950), PubMed (from 1965), and Cochrane (from 1991). Because mind-body exercise originated in the East, and China has the largest number of people practicing oriental mind-body exercise, this study also searched the CNKI. The end date of the consistent retrieval of this study is January 22, 2021. At the same time, the references included in the literature are searched manually, and the relevant literature is achieved by contacting the original author. This study uses 2 groups of keywords: mind-body exercise, Taijiquan, Baduanjin, and Qigong; blood pressure, essential hypertension, hypertension. Literature retrieval is conducted by two authors (LXH and CH) and confirmed by another collaborator (GBH) to ensure data retrieval accuracy.

We conducted this systematic review in accordance with the Guidelines for the Preferred Reporting Project (PRISMA)^[10]^ for systematic reviews and meta-analysis, and completed the research registration on the INPLASY platform (registration number: INPLASY202130072).

### 2.2 Inclusion and exclusion criteria

#### 2.2.1 Inclusion criteria

##### 2.2.1.1 Type of studies

We included randomized controlled trials (RCTs).^[11]^

##### 2.2.1.2 Type of participants

The experimental subjects included in this study are all hypertensive patients ≥45 years old.

##### 2.2.1.3 Type of interventions

In general, there are 2 ways to include the intervention subjects in this study: the intervention group simply adopted Mind-body exercise: the intervention group adopted Taijiquan or Baduanjin or Qigong, and the control group adopted other measures (mental health education or slow walking or sedentary or regular life or medicine, and so on) other than Mind-body exercise; the intervention group adopted the Mind-body exercise method plus other measures: the intervention group adopted Mind-body exercise (Taijiquan or Baduanjin or Qigong) plus other measures (mental health education or walking slowly or sedentary or regular life or medicine, and so on), the control group simply adopted other measures (mentioned above).

##### 2.2.1.4 Type of outcome measures

The purpose of this study is to measure the blood pressure of hypertensive patients. At this stage, the most intuitive way to evaluate blood pressure is to observe its systolic and diastolic blood pressure. Therefore, the main results of this study are: systolic blood pressure^[12]^; diastolic blood pressure.^[13]^

#### 2.2.2 Exclusion criteria

To complete this study accurately, this article has the following exclusion criteria:

(1) RCTs not in peer-reviewed journals;
(2) compared with the control group, Mind-body exercise is not the main factor in the intervention group measures;
(3) subjects are <45 years’ old, are not hypertensive or essential hypertension patients, or have a history of myocardial infarction or stroke;
(4) publication of meetings, publication of abstracts, reviews, publication of no detailed data, repeated publications, low-level academic literature, among others.

### 2.3 Risk of bias across studies

To evaluate the methodology of the included studies independently, the 2 authors (LXH and GBH) utilized the Physiotherapy Evidence Database (PEDro) scale,^[14]^ If there is any dispute, it will discuss with the third researcher (CH). The widely accepted methodological quality assessment tool includes 11 items: description of the inclusion conditions of the subjects; subjects are randomly assigned to each group; the mode of distribution is hidden; there is no significant difference in the baseline between the experimental group and the control group; all the subjects were blind; all the physiotherapists were blind; all the evaluators of at least one major result were blind; at least 85% of the subjects had major measurement results; all the participants were treated according to a randomly assigned scheme; the intragroup statistical results of at least one major result were reported; the study provided point measurements and variation measurements of at least one major result. However, during the actual mind-body intervention, it is not realistic to blind the participants in item 5 and the therapists in item 6. Therefore, these 2 items are not required in the quality evaluation of this study. In the end, there are 9 evaluation items, each of which is 1 point. If the evaluation literature meets the standard, the score will be 0.

### 2.4 Data collection process

Each article was extracted by 2 independent researchers (LXH and GBH), the third researcher (CH) confirmed, and transformed into 2 standard forms: descriptive data, including the first author and year of publication, research location and language, subjects’ health status, sample size, average age and age range, intervention schemes between the intervention group and the control group, and main measurement results; quantitative data, including the randomly assigned number of subjects, the mean ± standard deviation (SD) of the baseline data between the intervention group and the control group, and the mean ± standard deviation (SD) of the data after intervention between the intervention group and the control group.

### 2.5 Data synthesis and additional analyses

Stata 14.0 software was used to analysis the heterogeneity, sensitivity, and publication bias of all the outcome indicators included in the literature, and forest and funnel maps were drawn. Literature outcome indicators included in this articles belong to continuous variables, and the test units of each index are the same; therefore, the mean ± standard deviation (SD) is selected for statistics, and 95% CI is determined by the same time. The heterogeneity test was performed by *P* value and *I*^2^. If *P* > .10, there was no heterogeneity among the studies. If *P* < .10, there was heterogeneity among the studies. If *I*^2^ < 25%, then the heterogeneity between studies is considered small. If *I*^2^ > 50%, then the studies were considered to be noticeably heterogeneous. Subgroup analysis was utilized to explore the potential influencing factors of the outcome index of essential hypertension in the middle-aged and elderly.

## 3 Discussion

### 3.1 Summary of evidence

The research included in this review solely involved RCT studies that used combined effect size, subgroup analysis, sensitivity analysis, publication bias tests, and other methods to evaluate the effect and safety of mind-body exercise intervention on the blood pressure in middle-aged and elderly patients with hypertension.

### 3.2 Comparisons with previous studies

It has been proved that the quality of life of patients with hypertension is often worse than that of normal people.^[15]^ Most of the patients with hypertension are older people, which is a more economical and less side-effect treatment in addition to drug treatment. Reviewing previous studies, researchers on the therapeutic effects of Taijiquan or Qigong or Baduanjin on hypertension are mainly from China and South Korea. Chinese researchers believe that Taijiquan and Qigong may be supplementary and effective treatments for lowering blood pressure, but the therapeutic effect may be related to the increase of NO and the decrease of ET-1 in the blood.^[16]^ The researchers believe that Qigong is effective in the treatment of hypertension. In order to further verify the study's conclusions, it is necessary to design a higher intensity of evidence to prove it.^[17,18]^

However, the ages included in the 2 studies are different, 30 to 70 years’ old^[18]^ and 40 to 70 years’ old.^[17]^ It may be necessary to design RCT experiments to prove the therapeutic effect of people aged 30 to 40 years and to eliminate other possible biased factors. From the perspective of mechanism, researchers from South Korea believe that the effect of Qigong is not to reduce blood pressure directly but to stabilise the sympathetic nervous system and thus lower the blood pressure of patients. It is believed that Qigong is a complementary and irrational way to treat hypertension.^[19]^

## Acknowledgments

The authors acknowledge the participants for taking part in the study (Supplemental Digital Content: ).

## Author contributions

**Conceptualization:** Xianhui Liao, Beihai Ge.

**Data Curation:** Xianhui Liao, Beihai Ge, Hao Chen.

**Funding acquisition:** Beihai Ge.

**Funding:** Beihai Ge.

**Methodology:** Xianhui Liao, Beihai Ge, Hao Chen.

**Project Administration:** Xianhui Liao, Beihai Ge.

**Resources:** Xianhui Liao, Beihai Ge, Hao Chen.

**Supervision:** Xianhui Liao.

**Writing – original draft:** Beihai Ge, Hao Chen, Xianhui Liao.

**Writing – review & editing:** Beihai Ge, Hao Chen, Xianhui Liao.

## Supplementary Material

Supplemental Digital Content
