# Supplementary material for: The effect of mind-body exercise on blood pressure in middle-aged and elderly patients with hypertension: A protocol for a systematic review and meta-analysis
Source: Medicine (Baltimore). 2021 Jun 25;100(25):e26452. doi: 10.1097/MD.0000000000026452 (PMC8238318; doi:10.1097/MD.0000000000026452)
Supplement: Supplemental Digital Content [file medi-100-e26452-s001.docx]

**Search Strategy**

**Pubmed**

(blood pressure[MeSH] OR essential hypertension[MeSH] OR hypertension[MeSH]) AND (mind-body exercise[MeSH] OR taichi[MeSH] OR taijiquan[MeSH] OR baduanjin[MeSH] OR qigong[MeSH]）

FORm 1965

**Cochrane library**

#1: blood pressure[MeSH]

#2: essential hypertension[MeSH]

#3: hypertension[MeSH]

#4: mind-body exercise[MeSH]

#5: taichi[MeSH]

#6: taijiquan[MeSH]

#7: baduanjin[MeSH]

#8: qigong[MeSH]

(#1 OR #2 OR #3) AND (#4 OR #5 OR #6 OR #7 OR #8)

From 1991

**WOS (Web of science)**

TS = (blood pressure OR essential hypertension OR hypertension) AND TS = (mind-body exercise OR taichi OR taijiquan OR baduanjin OR qigong）

From 1950

**CNKI（China National Knowledge Infrastructure）**

SU = blood pressure + essential hypertension + hypertension AND SU = mind-body exercise + taichi + taijiquan + baduanjin + qigong

FORm 1979
